# Enhancing equity and efficiency in cervical screening uptake: a multidisciplinary quality improvement initiative

**DOI:** 10.1136/bmjoq-2024-003111

**Published:** 2025-08-11

**Authors:** Carlos Santos, Julie Roye, Joyce Tucker, Christina Guevara

**Affiliations:** 1 East London NHS Foundation Trust, London, UK

**Keywords:** Continuous quality improvement, Healthcare quality improvement, Health Equity, Quality improvement

## Abstract

**Background:**

Cervical cancer screening is vital for early detection and prevention, yet uptake remains suboptimal in diverse communities.

**Local problem:**

Cauldwell Medical Centre reported cervical screening uptake rates of 54% (ages 25–49) and 62% (ages 50–64) by June 2022, both significantly below the national target of 80%, with a concerning 8 percentage point disparity between age groups.

**Methods:**

Using quality improvement (QI) methodologies, including Plan-Do-Study-Act cycles and statistical process control charts, the team tested eight cycles of change grouped into three high-impact actions designed to improve accessibility, trust and personalisation of cervical screening services. Tests of change included culturally sensitive outreach, extended clinic hours and a self-booking system to enhance accessibility and engagement.

**Results:**

This QI initiative achieved a marked reduction in age-related inequalities in cervical screening uptake. By the end of the intervention period (March 2023), screening rates increased from 54% to 69% among women aged 25–49 and from 62% to 72% among women aged 50–64, narrowing the gap from 8 to 3 percentage points—a 60% reduction in disparity. By the final monitoring week, uptake further increased to 73% (ages 25–49) and 82% (ages 50–64), demonstrating how structured QI approaches can amplify the effectiveness of existing healthcare processes.

**Conclusions:**

This project highlights that systematically applying QI methodologies can effectively address healthcare inequalities, providing a scalable model for improving cervical screening uptake among under-represented populations.

**WHAT IS ALREADY KNOWN ON THIS TOPIC**

- Despite cervical cancer being largely preventable through regular screening, uptake rates remain suboptimal, particularly in deprived and diverse communities. Barriers such as cultural stigma, language challenges and accessibility issues contribute to health inequalities.

**WHAT THIS STUDY ADDS**

- This study demonstrates how applying quality improvement (QI) methods, including the Model for Improvement and iterative Plan-Do-Study-Act cycles, can drive measurable improvement in cervical screening uptake. It highlights the role of data-driven decision-making, stakeholder co-design and small tests of change in addressing barriers and reducing health inequities.

**HOW THIS STUDY MIGHT AFFECT RESEARCH, PRACTICE OR POLICY**

- By leveraging QI principles, this work offers a scalable and sustainable model for enhancing preventive care services. It emphasises the importance of real-time measurement and continuous learning in achieving health equity, providing insights for healthcare systems seeking to improve population health outcomes through structured improvement efforts.

## Problem

 Cervical cancer remains a significant public health concern, with disparities in early detection and prevention contributing to avoidable mortality. Healthcare organisations play a pivotal role in tackling these inequalities through preventive care. The East London NHS Foundation Trust (ELFT) serves a population of 1.8 million people, characterised by cultural diversity and a variety of social needs, including differing levels of income, education and access to healthcare services. ELFT offers a wide range of healthcare services, including mental health, community health and primary care. As part of ELFT, Cauldwell Medical Centre (CMC) launched a quality improvement (QI) initiative to address gaps in its cervical screening service in Bedfordshire, where screening rates were 54% (ages 25–49) and 62% (ages 50–64) as of June 2022, with an 8 percentage point disparity between age groups. ELFT’s commitment to patient safety and continuous improvement^1^,^7^ provided a strong foundation to this effort, with a particular focus on improving accessibility and awareness among non-English-speaking communities.

Several interconnected factors contributed to unequal screening access. Using an Ishikawa Fishbone diagram,^8^ the team identified key barriers: limited awareness, cultural beliefs, language barriers and mistrust in the healthcare system. These challenges were particularly pronounced among postmenopausal women, individuals with disabilities, diverse ethnic backgrounds and the transgender community. For clarity, the term ‘women’ in this paper refers to all persons with a cervix and uterus, recognising that not all such individuals identify as female.

Embarrassment, mistrust and discomfort related to cervical smears further deterred participation. Additionally, structural barriers such as limited accessibility in deprived areas and marginalised communities required targeted initiatives to rebuild trust, enhance communication and improve access.

Procrastination and limited understanding of cervical screening added another layer of complexity. Addressing these barriers involved fostering informed decision-making through culturally sensitive and targeted educational approaches.

This comprehensive understanding laid the foundation for testing changes aimed at improving service delivery and reducing health inequalities at CMC, with a specific aim to increase cervical screening uptake at CMC to 80% by March 2023.

## Background

Cervical cancer, a critical global health issue, impacts millions of women worldwide regardless of their ethnic or socioeconomic backgrounds.^8^ Despite the availability of effective screening options, it remains a prominent cause of cancer-related deaths.^8^ As such, it is essential to amplify the awareness of cervical cancer screening, targeting diverse groups including menopausal individuals, trans men and those with learning disabilities.^9^,^12^

In England, the National Health Service (NHS) has been vocal in encouraging all eligible individuals to prioritise cervical screening, underlining its potential to save lives.^13^ However, recent statistics show that close to one-third of eligible individuals, approximately 4.6 million, have not taken advantage of the screening opportunities.^14^ This underutilisation can be attributed to factors such as embarrassment and misconceptions, especially among lesbian and bisexual women.^15^

Cervical cancer thus remains a significant public health concern in England, with nearly 2700 new cases annually and about 690 resultant deaths every year, translating to roughly two deaths daily.^12^ Although screening can prevent up to 70% of cervical cancer deaths, attendance rates remain below optimal levels, with predictions indicating that regular screening could prevent as many as 83% of deaths.^16^

In terms of primary prevention, the NHS introduced the human papillomavirus (HPV) vaccine in 2008 as part of its routine vaccination schedule, significantly reducing the incidence of cervical cancer: a study published in *The Lancet* estimated that the HPV vaccination programme prevented approximately 450 cases of cervical cancer and 17 200 cases of cervical carcinoma in situ by mid-2019.^17^

Recent innovations in screening methods include HPV self-sampling, which has shown promise in increasing uptake among underscreened populations.^18^ Self-sampling allows individuals to collect their own samples, which can then be sent to a laboratory for analysis. This method has been associated with improved participation in cervical cancer screening.^19^

Certain regions, such as Bedford, have cervical screening uptake rates below the national average—58.3% compared with 80%.^20^ Identified obstacles to screening such as lack of knowledge, cultural beliefs, language barriers and mistrust of healthcare providers^21^ necessitate targeted interventions to boost uptake.

Therefore, a multifaceted strategy is crucial for increasing uptake and minimising health disparities. This involves catering to diverse patient needs through improved access, multilingual educational materials and community involvement.^13^ Primary healthcare providers are central to this effort, providing education, counselling, referrals and collaborating with community organisations to create culturally sensitive resources,^22^ ultimately saving lives and improving women’s health across the UK. Sustained awareness campaigns and joint action are fundamental in the ongoing battle against cervical cancer and health disparities.

## Measurement

To establish a baseline understanding of cervical screening uptake at CMC, we analysed electronic health record data from 2021 to 2022. This initial assessment revealed a significant disparity in uptake between age groups, with 62% of women aged 50–64 participating in screening, but only 54% of women aged 25–49 as of June 2022. The objective of this QI project was to narrow this disparity and improve the overall screening uptake towards the 80% national target. Baseline figures represent screening uptake rates at project initiation (June 2022), while endpoint figures represent final rates at project conclusion (March 2023). The following measures were defined to track the project impact:

### Outcome measures

- *Percentage of women screened*: recorded weekly, measuring the percentage of eligible women completing cervical screening. This measure tracked overall intervention effectiveness and progress towards the 80% target using statistical process control (SPC) charts.
- *Age group comparison***:** tracked weekly, measuring screening rates for women aged 25–49 and 50–64. This measure evaluated the reduction of age-related disparities, focusing on the under-represented 25–49 group.

### Process measures

- *Appointment bookings*: tracked weekly, measuring the number of eligible women booking cervical screening appointments after receiving invitations. This assessed the effectiveness of communication and outreach strategies.
- *Appointment attendance*: tracked weekly, measuring the number of eligible women attending their scheduled appointments. This evaluated how effectively initial interest translated into actual participation.
- *Attendance rate*: tracked weekly, measuring the percentage of booked appointments attended. This provided a comprehensive view of the intervention’s success in driving participation.

### Balancing measures

- *Declined invitations*: tracked weekly, recording the number of patients declining invitations and their reasons. This ensured improvements did not cause unintended negative effects, such as anxiety or dissatisfaction.
- *Healthcare staff workload*: monitored weekly, assessing workload through feedback sessions and tracking appointment volumes. This measured the impact of changes on staff capacity and resource strain.
- *Patient satisfaction*: collected routinely, capturing feedback on the new process to identify any unintended consequences and ensure a positive patient experience.

## Design

Recognising the need for collaboration, a multidisciplinary QI team was established, comprising general practitioners (GPs), practice nurses, pharmacists, administrative staff and, crucially, patient representatives. Supported by a senior sponsor and a QI coach throughout the project, the team met every 2 weeks to review progress, share insights and plan subsequent steps. Active patient involvement permeated the project from inception, with their insights and contributions shaping the tests of change and communication materials to better resonate with specific populations.

Guided by the Model for Improvement^23^ and ELFT’s Sequence of Improvement,^4^ the team followed a methodology involving five critical steps: identifying the quality issue, understanding the problem, developing a strategy, testing change ideas and implementing successful changes—a structured technique that has been consistently applied for over a decade.^1^,^7^

**Figure 1:**
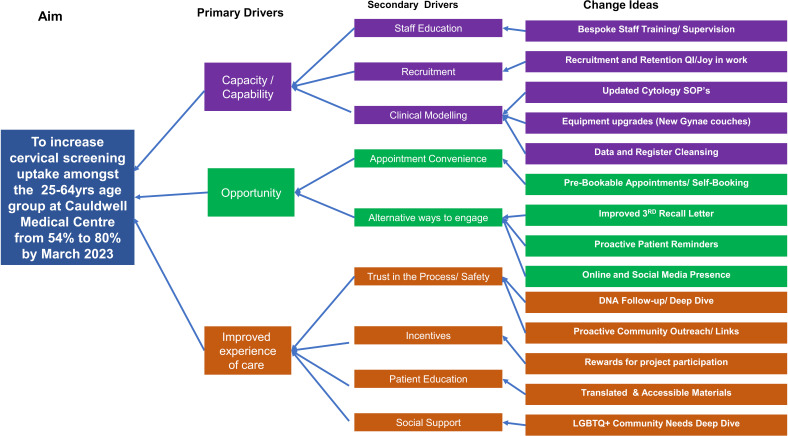
Driver diagram illustrating the aim, primary and secondary drivers, and associated change ideas designed to increase cervical screening uptake among 25–64 years old at Cauldwell Medical Centre. The project focused on improving capacity, opportunity and patient experience to drive sustainable change. QI, quality improvement; SOP, Standard Operating Procedure.

To gain a deeper understanding of factors affecting cervical screening uptake, the team conducted an Ishikawa Analysis,^23 24^ identifying key barriers at individual, practice and community levels. Barriers included limited awareness and misconceptions about screening, language barriers and mistrust in the healthcare system. The analysis, conducted using a hybrid of face-to-face and videoconferencing meetings, enabled all team members to contribute their insights. These findings were summarised and informed the development of a driver diagram (figure 1), which outlined the essential components required to achieve the intended outcomes.^25^

## Strategy

A Plan-Do-Study-Act (PDSA) approach underpinned the improvement strategy, enabling the team to iteratively test, refine and scale up change ideas in response to real-time feedback and evolving insights. Rather than working through fixed categories, the team pursued three high-impact actions—each addressing multiple aspects of the cervical screening pathway. These actions consisted of eight interrelated cycles of change, progressing cumulatively to deliver improved outcomes.

**Figure 2:**
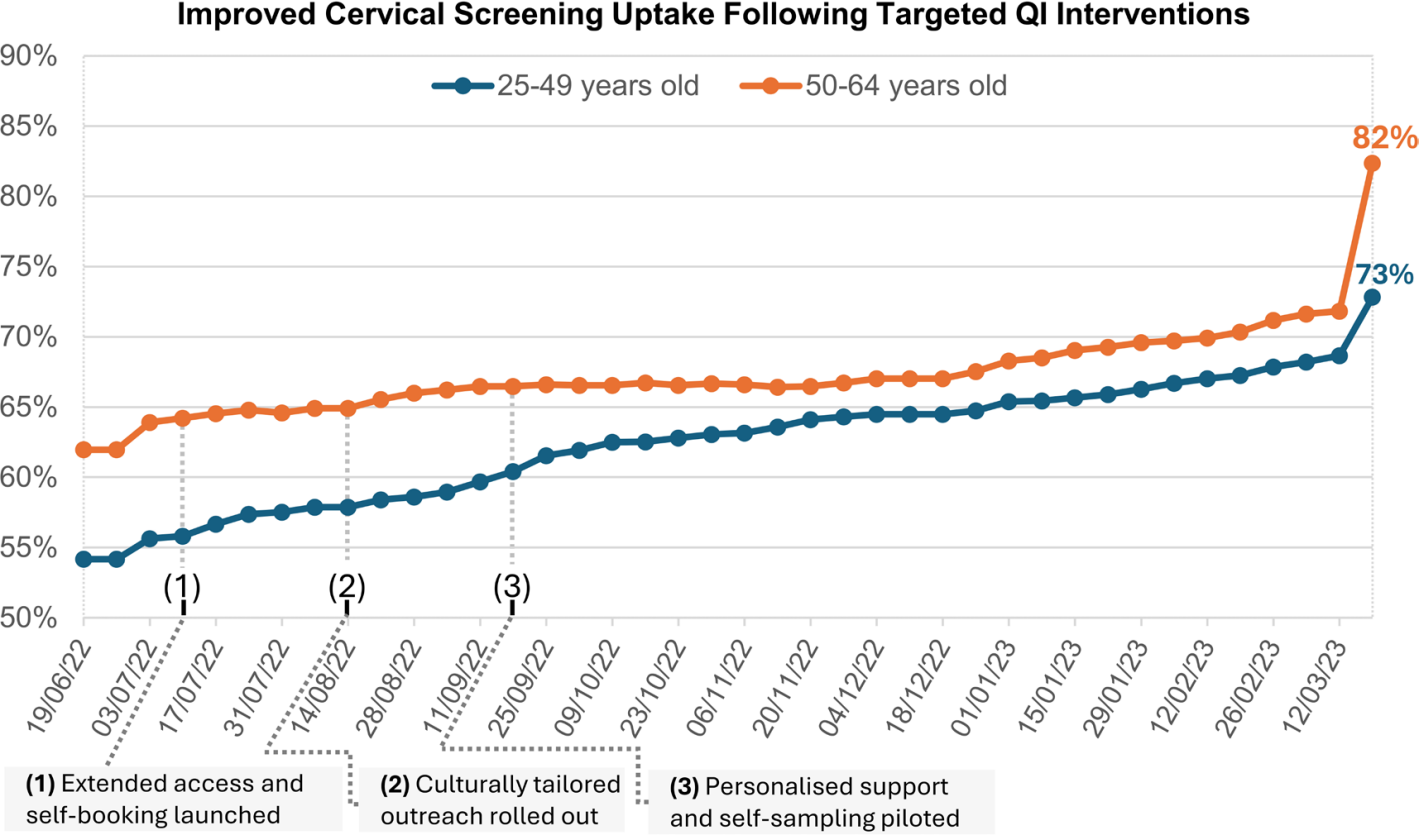
Line chart showing the improved percentage of cervical screening uptake at Cauldwell Medical Centre, segmented by age group. Following the launch of extended access clinics and self-booking (1), culturally tailored outreach (2) and piloting of personalised support and self-sampling (3), uptake increased across both age groups, with a sustained narrowing of the gap between cohorts in the postintervention period. QI, quality improvement.

The first high-impact action, started in July 2022 (figure 2), focused on enhancing accessibility. In its initial cycle, the team introduced extended clinic hours through evening appointments, aiming to accommodate patients constrained by work, family or caring responsibilities. In a subsequent cycle, a self-booking system was implemented to improve autonomy and convenience for patients navigating their appointments. While these adjustments yielded a modest increase in bookings, process data revealed that certain groups still failed to engage, signalling deeper needs around trust and communication.

The second high-impact action, initiated in August 2022, concentrated on establishing trust and fostering inclusive communication. In its first cycle, the team developed culturally sensitive, easy-read leaflets, co-designed with patients, to demystify the screening process and articulate its benefits. A second cycle further refined these materials based on patient feedback and frontline staff input. A third cycle introduced personalised invitations from known GPs and involved community connectors to support outreach among historically underserved groups. These changes resonated with many patients, who described a greater sense of safety and familiarity—yet it became evident that additional personalisation would be required for individuals with specific vulnerabilities.

Finally, in September 2022, the third high-impact action aimed at delivering personalised, trauma-informed care. In its first cycle, healthcare professionals received training in emotional safety and trauma sensitivity, equipping them to better support patients during consultations. A second cycle clarified internal referral pathways to ensure swift access to specialist input when needed, reducing procedural uncertainty. In a final cycle, a self-sampling option was piloted, offering patients a more private and flexible alternative to traditional cervical screening methods.

To establish the impact of each change idea, data over time was displayed using SPC charts—a key tool in healthcare for understanding variation in systems and identifying improvements based on standardised SPC rules.^26 27^ Regular case discussions and staff reflections enabled the team to adapt quickly, ensure fidelity to the change theory and maintain momentum. Rather than discrete, isolated tests, the cycles of change were explicitly connected—each building on the lessons from previous cycles to progressively reduce inequalities in access, experience and outcomes.

## Results

### Outcome measures

The primary outcome was a reduction in disparity of cervical screening uptake between women aged 25–49 and 50–64. Weekly monitoring (figure 2) revealed substantial progress in narrowing this gap:

- *Baseline disparity (June 2022):* 8 percentage points (54% vs 62%)
- *End-of-intervention disparity (March 2023):* 3 percentage points (69% vs 72%)
- *Gap reduction achieved:* 5 percentage points, representing a 60% reduction in age-related disparity

During the final monitoring week, rates further increased to 73% (25–49) and 82% (50–64), demonstrating how QI can amplify the effectiveness of routine healthcare processes.

### Process measures

**Figure 3:**
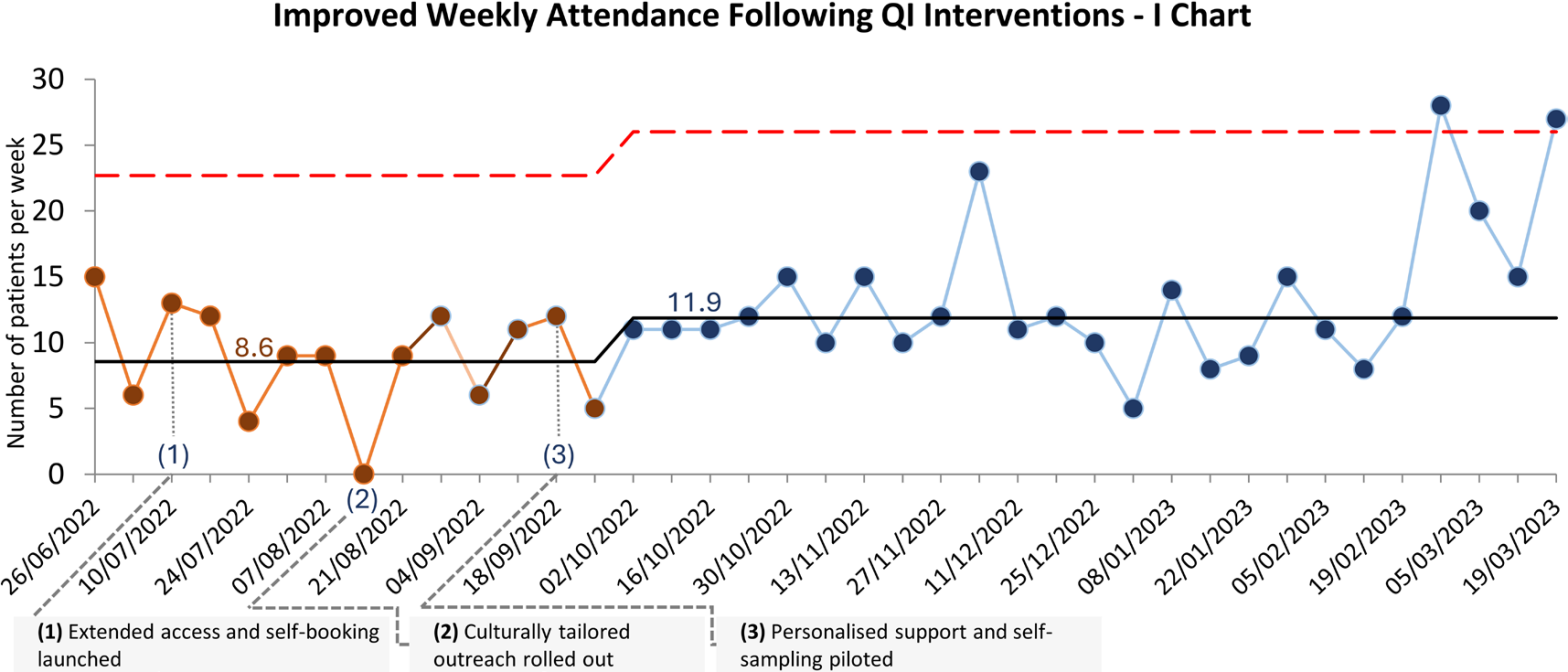
An Individuals (I) chart displaying the number of patients attending cervical screening appointments per week at Cauldwell Medical Centre. Following the introduction of three high impact actions—(1) extended access and self-booking, (2) culturally tailored outreach and (3) personalised support with self-sampling—weekly attendance increased, with a sustained shift above the baseline average observed. QI, quality improvement.

Process measures captured the effectiveness of communication and outreach strategies in driving participation:

- Weekly booked appointments increased from an average of 11–18.
- Attendance at scheduled appointments (figure 3) improved from an average of 9–12.

These improvements reflected the success of targeted outreach efforts in translating initial interest into actionable participation.

### Balancing measures

Balancing measures ensured that the tests of change did not inadvertently cause negative consequences. Although specific quantitative data on these measures was not systematically collected, we focused on several key areas to monitor potential adverse effects:

- *Healthcare staff workload:* regular feedback sessions tracked staff workload, confirming that changes did not overburden the team.
- *Patient satisfaction:* qualitative feedback indicated positive reception, reinforcing the importance of patient-centred care.

By maintaining focus on these areas, the team ensured that positive outcomes in screening uptake were achieved without compromising patient or staff well-being.

## Discussion

This QI initiative successfully demonstrated the effectiveness of targeted interventions in improving cervical screening uptake. By employing PDSA cycles and iteratively refining change ideas based on real-time feedback, the project achieved meaningful progress toward the national target of 80% screening uptake.

The most significant achievement was the 60% reduction in age-related screening disparities, from 8 to 3 percentage points by March 2023 (figure 2). This demonstrates that targeted QI can effectively address healthcare inequities when systematically applied. The acceleration observed during the final monitoring period, reaching rates of 73% and 82% (figure 2), occurred during routine healthcare quality monitoring cycles,^28^ which typically drive intensified focus on preventive care services across primary care settings. This pattern suggests that improvements in accessibility, trust and personalisation can create conditions for accelerated uptake when combined with routine healthcare system processes.

The cumulative impact of interventions was particularly notable. While each PDSA phase introduced distinct strategies—expanded appointment availability, culturally sensitive educational materials and a self-sampling programme—their cumulative effect became apparent only after all components were implemented. Attempting to implement these strategies simultaneously would likely have overwhelmed both patients and staff, leading to inefficiencies and limited uptake. The PDSA approach was instrumental in breaking down strategies into manageable, testable cycles, allowing the team to refine change ideas in real time and address challenges as they arose. This iterative process ensured that the interventions complemented each other, fostering a seamless and patient-centred experience.

Several elements contributed to the project’s success. Expanding access through evening clinics and online self-booking improved convenience, particularly for patients with work or family commitments. Culturally sensitive educational materials and personalised invitations built trust and addressed communication barriers, particularly among non-English-speaking populations—aligning with existing research on the effectiveness of targeted education in improving health outcomes.^29^,^32^ Additionally, the self-sampling programme offered a flexible, less invasive screening option, reducing procedural hesitancy and enhancing patient autonomy. These interventions collectively narrowed the gap in screening rates between age groups while increasing overall participation.

The increase in participation rates observed through time (figures2  3) underscores the importance of allowing interventions to consolidate over time. This surge in engagement reflects the cumulative effect of consistent messaging, accessibility improvements and trust-building measures rather than any additional intervention. These findings highlight the value of a structured, phased strategy to tackle complex healthcare challenges.

## Lessons and limitations

This cervical screening project provided several valuable lessons while revealing important limitations that offer insights for future initiatives.

1. *Reaching vulnerable groups:* engaging marginalised communities remains challenging, with persistent barriers such as cultural resistance and accessibility issues. Collaborating with community leaders and faith groups can help build trust and promote participation.
2. *Understanding non-attendance:* data limitations prevented a detailed understanding of why some individuals did not attend screenings. Collecting comprehensive data on non-attendance is critical for tailoring interventions to address specific barriers.
3. *Generalisability:* the findings are context-specific, reflecting the demographics and cultural nuances of the local population. Adapting these interventions to other settings requires careful consideration of local differences.
4. *Balancing measure data:* the absence of quantitative balancing measures, such as staff workload and patient satisfaction, limits a comprehensive evaluation of the project’s broader impact. Future studies should incorporate robust data collection methods to fill these gaps.
5. *Seasonal and system factors*: the project timeline encompassed routine healthcare quality cycles^28^ that may have influenced final outcomes. The acceleration in uptake during the final monitoring period highlights how QI interventions can amplify the effectiveness of existing system processes. Future projects should consider these cyclical factors when planning intervention timelines and interpreting outcomes to distinguish between intervention effects and organisation-wide performance drivers.

## Conclusion

This QI initiative successfully reduced age-related disparities in cervical screening uptake by 60% over the 9-month intervention period, narrowing the gap from 8 to 3 percentage points (figure 2) through systematic interventions addressing accessibility, trust and personalisation. Final screening rates of 69% (25–49) and 72% (50–64) by the end of the intervention period, with further increases to 73% and 82% respectively during the final monitoring week, demonstrate both the sustained reduction of inequity and the potential for QI interventions to enhance overall preventive care access.

To ensure sustainability, successful interventions have been embedded into routine practice, with ongoing monitoring and patient feedback supporting continuous improvement. Future efforts will focus on enhancing data collection to assess the long-term sustainability of these interventions and their ongoing impact on screening uptake, to better understand non-attendance and to inform targeted outreach strategies. This project provides a robust foundation for scaling patient-centred interventions to improve preventive healthcare services and reduce disparities.

## Data Availability

Data are available upon reasonable request.
